# Development of *Cystoisospora felis* in Cell Culture and *in vitro* Formation of Monozoic Tissue Cysts

**DOI:** 10.3389/fvets.2019.00361

**Published:** 2019-10-16

**Authors:** Rachel Ferreira, Waléria Borges-Silva, Rogério F. de Jesus, Luís F. P. Gondim

**Affiliations:** Department of Veterinary Anatomy, Pathology and Clinics, School of Veterinary Medicine and Animal Science, Federal University of Bahia, Salvador, Brazil

**Keywords:** *Isospora felis*, *Cystoisospora* sp., tissue cyst, sporozoite, oocyst

## Abstract

*Cystoisospora felis* is a coccidian parasite commonly found in feces of domestic cats. Infection in cats occurs by ingestion of sporulated oocysts or consumption of rodents infected by the parasite. Scarce information is available about extraintestinal stages of *C. felis* in naturally infected intermediate hosts, as well as in cell culture. The aim of the current work was to investigate the development of *C. felis* in Vero cells (African green monkey kidney) and MDCK cells (Madin-Darby canine kidney). Cell monolayers were inoculated with mechanically released sporozoites of *C. felis*, and parasite growth was daily examined using light microscopy. After cell invasion, only parasitophorous vacuoles containing a single zoite were observed. Five days post-inoculation with sporozoites, unstained cell monolayers were evaluated by differential interference contrast (DIC), and also by Romanovsky stain using conventional light microscopy. Single zoites, each surrounded by a cyst wall, were observed by both methods. Multiplication by endodyogeny did not occur in any cell monolayer. Treatment of encysted parasites with HCl-pepsin for 15 min led to dissolution of the cyst wall and release of intact and motile zoites. To our knowledge, this is the first demonstration of *in vitro* production of monozoic tissue cysts of *C. felis*. As kittens commonly shed *C. felis* in their feces, oocysts are easily available for *in vitro* production of monozoic tissue cysts of the parasite. Development of *C. felis* in cell culture may be employed as a model on tissue cyst formation of *Cystoisospora* spp. and closely related coccidia.

## Introduction

*Cystoisospora felis* (syn *Isospora felis*) is a coccidian parasite of the family Sarcocystidae, which is frequently found in feces of domestic cats worldwide (1, 2). Identification of *C. felis* is easily performed by visualization of its large ovoid oocysts in fecal specimens that measure 32–53 × 26–43 μm (3). After shedding non-sporulated oocysts in the environment, sporulation takes place in a few hours after excretion of oocysts (4). Before 1970, cats were considered the only host of the parasite with an oral-fecal life cycle, but later on, tissue cysts were described in rodent hosts (2). Intermediate hosts become infected by ingestion of sporulated oocysts in the environment and may harbor extraintestinal tissue cysts of the parasite (1).

Development of several cyst-forming coccidia that use avian and mammalian intermediate hosts has been described for decades in a broad variety of cell lineages using excysted sporozoites as infective material (5, 6). However, information regarding extraintestinal stages of *Cystoisospora* spp. in cell culture is scarce (7). In the present work, we aimed to culture *C. felis* in two cell lineages and characterize the development of parasite stages observed in cell culture.

## Materials and Methods

### Obtention and Sporulation of *C. felis* Oocysts

Ten 20-day-old cats were acquired from an animal shelter, located in the city of Salvador, Bahia. The animals were maintained in the animal facility of the Veterinary Hospital at the Federal University of Bahia. Cats were placed in two metal cages with water and food *ad libitum* and at an average temperature of 26°C. The cages were enriched with toys and soft bedding.

The cats showed natural infections by *C. felis* and pools of their feces were collected daily and examined for 30 days by a conventional sucrose-centrifugation (sucrose density = 1.25 g/ml) technique. The total volume of feces in 24 h was homogenized and screened for oocysts. Five grams of feces were mixed with water, filtered using gauze, placed in 50 ml tubes, centrifuged at 1,200 g for 10 min, and the sediment mixed with sucrose solution in 15 ml tube. This suspension was centrifuged at 1,200 g for 10 min and ~50 μl of the supernatant was placed on a glass slide with coverslip for microscopical examination. Fecal samples containing *C. felis* oocysts were concentrated by sucrose flotation at the same day oocysts were found. Oocysts were washed in distilled water to remove sucrose, and the sediment suspended in 2% potassium dichromate (w/v) for sporulation in an Erlenmeyer on an automated mixer during 5 days (8, 9). Species identification as *C. felis* was confirmed by visualization of oocysts containing two sporocysts each and measurement of oocysts. Oocysts presenting lengths equal or superior to 32 μm and absence of other smaller oocysts (length <32 μm) were saved for posterior procedures.

### *In vitro* Production of *C. felis*

#### Excystation of Sporozoites

Sporulated oocysts maintained in 2% potassium dichromate at 4°C were washed three times in distilled water (1,200 g, 10 min at 4°C) to remove potassium dichromate. The excystation procedure for *C. felis* was performed similarly as described for oocysts of *Hammondia heydorni* (10). An aliquot containing 1 × 10^6^ oocysts was suspended in 1 ml of water, placed in a 1.5-ml plastic tube, and centrifuged (1,200 g at 4°C) for 10 min. A sodium hypochlorite solution (2% of active chlorine) was added to the sediment to a 1 ml volume and the solution was incubated for 30 min under continuous agitation. The suspension was washed five times in RPMI medium (1,200 g for 10 min at 4°C) and the resultant sediment mixed with 0.5 ml RPMI. A 1.5-ml tube was filled with glass beads (425–600 μm, Sigma-Aldrich Brasil Ltd., São Paulo, Brazil) to a volume of 400 μl. The glass beads were incubated with sodium hypochlorite for 30 min under continuous agitation and washed five times in RPMI (1,200 g for 10 min at 4°C). The 0.5 ml solution containing the oocysts was transferred into the 1.5-ml tube with glass beads and vortexed for 90 s. A 10 μl suspension was aseptically removed from the tube and placed on a glass slide for microscopic observation of released sporozoites. The remaining solution (~0.5 ml) containing a mixture of released sporozoites, and fragmented oocyst walls and sporocyst walls, were saved for the next step.

#### Cultivation of *C. felis* in Vero and MDCK Cell Lines

A 0.5 ml suspension of lysed *C. felis* oocysts, where intact sporozoites were visualized, was added to a monolayer of Vero cells (African green monkey kidney, ATCC® CCL-81™) in RPMI containing 5% FCS and 1% antibiotic/antimycotic (100 U penicillin, 100 μg streptomycin/ml, and 0.25 μg/ml of amphotericin B). The same amount of lysed *C. felis* oocysts was added to a monolayer of MDCK (Madin-Darby canine kidney, ATCC® CRL-2936™) cells. The cells were maintained in an incubator at 37°C with 5% CO_2_. The medium was changed every 2 days. In addition to use of a whole lysate of oocysts containing released sporozoites, Vero cells were also inoculated with purified sporozoites. Ruptured oocyst solution (1 ml) was passed through a sterile PD-10 Sephadex column (Pharmacia Biotech, Uppsala, Sweden); this method has been employed for *S. neurona* merozoites purification (11). Purified sporozoites were inoculated into a 25 cm^2^ flask containing a monolayer uninfected Vero cells.

Infected Vero cells in a 25 cm^2^ flask were transferred to a 24-well-culture plate by trypsinization. The medium of the 25 cm^2^ flask was removed and the monolayer was incubated for 5 min with 1 ml of a 0.25% trypsin solution (1×) with EDTA. The trypsin activity was blocked with RPMI and the content of the flask was distributed in the wells (12).

Excystation and inoculation of *C. felis* in Vero cells have been repeated three times using the whole lisate of oocysts in the cell monolayers. Additional two attempts were performed employing sporozoites filtered in Sephadex collumn. Oocysts were kept in 2% potassium dichromate at 4°C and used in excystation procedures between 30 and 180 days.

#### Treatment of Monozoic Tissue Cysts of *C. felis* With Acid Pepsin

Monozoic tissue cysts of *C. felis* in Vero cells on the fifth day after inoculation (DAI) were washed with RPMI and incubated with HCl-pepsin (0.5 g of pepsin, 0.5 g of NaCl, 98.6 ml of ddH_2_0, 1.4 ml of HCl, pH = 0.9) for 15 min (12). The acid pepsin grossly mimics the gastric content of monogastric mammalian hosts. After incubation with HCl-pepsin, the solution was washed two times in RPMI (350 g, 5 min). The sediment was resuspended in 1 ml RPMI and distributed on monolayers of non-infected Vero cells on 24-well culture plates. The Vero cell monolayers were grown on glass coverslips inside each well of the plate.

#### Light Microscopy and Differential Interference Contrast Microscopy (DIC)

Vero cell monolayers were grown on 25 cm^2^ culture flasks and infected with sporozoites of *C. felis*. On the fifth DAI, the bottom of the flask was cut and its internal surface fixed with methanol and stained by a commercial hematological stain (Panótico Rápido—Laborclin®, Pinhais, PR, Brazil). The observed parasitophorous vacuoles and zoites were measured using the software NIS—Elements, version 4.60 (NIKON®).

Differential interference contrast microscopy (DIC), using a Leica microscope (model TCS SP8), was performed in the Gonçalo Moniz Research Center at Oswaldo Cruz Foundation in Bahia, Brazil. Vero cells (4 × 10^4^ cells per well) were cultured in 24-well plates containing a glass coverslip of 12 mm diameter in each well. Oocysts (1 × 10^5^) were lysed as described above, and ~21 μl of the solution containing *C. felis* released sporozoites were placed in each well of the plate. The culture was daily observed and maintained for 5 days. Images obtained by DIC corresponded to *C. felis* on the fifth DAI.

## Results

### Sporozoites in Cell Culture and Treatment With Trypsin and Acid Pepsin

Excysted sporozoites derived from oocysts stored for different periods of time (between 30 and 180 days) were used for cell inoculations. *Cystoisospora felis* bisporocystic oocysts of average length of 39.6 μm, were mechanically disrupted by vortexing the oocyst solution in 1.5-ml plastic tubes filled with glass beads. More than 50% of sporozoites were excysted by vortexing 5 × 10^5^ oocysts with glass beads for 1 min.

Aging of oocysts did not inhibit their infectivity. After mechanical lysis, free motile sporozoites were visualized on cell monolayers. No infected MDCK cells were observed after inoculation of *C. felis* sporozoites. In contrast to MDCK cells, intracytoplasmatic zoites were visualized in Vero cells. For this reason, MDCK cells were no longer used in the experiment.

**Figure 1:**
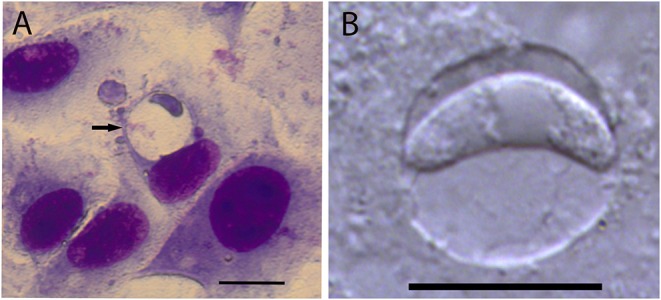
Monozoic tissue cysts of *Cystoisospora felis* developed in Vero cells at 5 days after inoculation with sporozoites. **(A)** A round cyst containing a visible wall (arrow) and a single zoite on its periphery. Quick hematological stain (Panótico Rápido). Bar = 20 μm. **(B)** An unstained monozoic cyst visualized by differential interference contrast microscopy (DIC). Bar = 10 μm.

**Figure 2:**
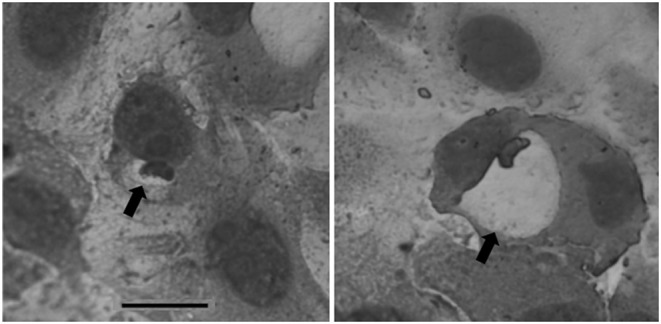
*In vitro* produced monozoic tissue cysts of *Cystoisospora felis* (arrows) presenting distinct diameters. Quick hematological stain (Panótico Rápido). Image captured using a monochromatic filter. Bar = 25 μm.

At 24 h after infection, single sporozoites were observed in a small proportion of Vero cells. At 3 DAI, parasitophorous vacuoles (PV) became enlarged and contained a single zoite. At 5 DAI, parasitophorous vacuoles became even larger and with thicker walls, however, thickness of the walls were not determined (Figure 1). PV possessed variation in size with zoites located on their periphery of the vacuole (Figure 2). Measurements of PV and individual zoites are presented in Table 1. No multiplication by endodyogeny or the presence of other parasite stages occurred during cell culture. PV could be visualized in cell culture until 7 DAI, however, in cultures maintained for more than 7 days, visualization of PV was impaired by the growth of Vero cells that supplanted the original cell monolayer.

**Table 1:** Measurements (in micrometers) of monozoic tissue cysts of *Cystoisospora felis* (*n* = 27) 5 days after inoculation with mechanically released sporocysts in cell culture.

	**Amplitude**	**Minimal size**	**Maximum size**	**Mean**	**Standard deviation**
Length	25.96	12.59	38.55	25.49	6.08
Width	22.82	7.88	30.70	20.92	5.60
Zoite's length	6.95	6.85	13.80	10.22	1.73
Zoite's width	3.29	3.53	6.82	4.95	0.78

Vero cells containing PV of *C. felis* were treated with trypsin and transferred to 24-well culture plates containing Vero cell monolayers on glass coverslips. Treatment with trypsin for 5 min did not affect integrity of PV in Vero cells, however, lower number of PV were retrieved as a result of cell manipulation, as some cells were probably damaged during this procedure.

Cells infected with *C. felis* at 5 DAI were treated for 15 min with HCl-pepsin. After treatment, motile zoites were observed in the supernatant and associated PV were dissolved by the acid solution. Besides the observed motility of released zoites, no PV was observed on Vero cell monolayers.

## Discussion

In the current study, *C. felis* oocysts were obtained from naturally infected cats and sporulated in 2% sodium dichromate. After storing in the same dichromate solution for a maximum of 6 months, oocysts were mechanically disrupted and used for cell inoculations. Two types of sporozoite inocula were employed in the experiment. The first one was obtained by disruption of oocysts with glass beads and inoculation of the whole lysate in Vero cells. The second inoculum consisted of disrupted oocysts, which were filtered using sterile Sephadex collumn, and inoculation of purified sporozoites in cell monolayers. In both types of inocula, *C. felis* sporozoites invaded cells and cell monolayers remained intact. In a previous work (10), *Hammondia heydorni* oocysts were disrupted solely by mechanical lysis and a reasonable number of sporozoites were infective to cell monolayers. The use of chemical compounds for excystation of sporozoites, such as bile products (7, 13), probably lead to a greater number of excysted parasites, but the single use of mechanical disruption seems to be a practical and efficient excystation method.

In a previous work, *Cystoisospora belli* oocysts were obtained from HIV-infected human patients, and sporozoites cultured in four cell lines (14). These authors reported that Vero cells presented the best growth of *C. belli* merozoites, which were shown to divide by endodyogeny in this cell type. In the present work, Vero cells were selected as host cells for *C. felis* development *in vitro*, but multiplication by endodyogeny did not occur after inoculation of *C. felis* sporozoites in Vero cells. Our work contrasts to a study developed in the 1970s (15), whose authors reported multiplication by endodyogeny for *C. felis* in different cell lines. Interestingly, *Cystoisospora canis* had also been reported to multiply by endodyogeny in cell culture using different cell types (16). However, a few decades later, *C. canis* sporozoites were cultured in several cell lines and in all of them no multiplication by endodyogeny was observed for this parasite (7, 12). It is possible that in the two *in vitro* studies on *C. felis* and *C. canis* performed in the 1970s (15, 16) there was some degree of contamination with oocysts from other coccidia (*Hammondia* spp., *Toxoplasma gondii, Neospora caninum*), which are known to multiply by endodyogeny in cell culture (17–20). Another possible explanation for the reported multiplication by endodyogeny for *C. canis* and *C. felis*, as already raised by Houk and Lindsay (12) for the *C. canis* study, is the presence of *Cystoisospora* spp. strains with different biological behavior.

In our current work, *C. felis* sporozoites developed into monozoic tissue cysts in Vero cells. The resistance of zoites to HCl-pepsin treatment and the morphology of VP, characterized by a single zoite surrounded by a cyst wall, are compatible with monozoic tissue cysts reported for *Cystoisospora* spp. in animal host and cell culture (7, 12, 14, 21).

The confirmation of this parasite stage relied on its morphology, characterized by a single zoite surrounded by a cyst wall, absence of replication by endodyogeny, as well as the resistance of the zoite to HCl-pepsin treatment. We have attempted to examine *in vitro* produced monozoic tissue cysts by transmission electron microscopy, however, quality of images was poor and did not allow appropriate observations. To the best of our knowledge, this is the first demonstration of monozoic tissue cysts of *C. felis* in cell culture. It is interesting to note that the largest *Cystoisospora* spp. oocysts shed by cats and dogs correspond to *C. felis* and *C. canis*, respectively, which turned out to have similar *in vitro* development in monkey kidney cells. As *C. felis* is frequently shed by naturally infected cats, oocysts are easily available for *in vitro* production of monozoic tissue cysts of the parasite. Comparative studies using *in vitro* produced cysts of this parasite may favor further works on mechanisms involved in tissue cyst formation of *Cystoisospora* spp. and in closely related coccidia.

## Data Availability Statement

The datasets generated for this study are available on request to the corresponding author.

## Ethics Statement

The animal study was reviewed and approved by Ethical Committee for Animal Experimentation of the School of Veterinary Medicine, at the Federal University of Bahia (Protocol 06/2018). After the conclusion of the experiment, all cats were surgically spayed at the Veterinary Hospital (Federal University of Bahia) and adopted by veterinary students.

## Author Contributions

RF: animal care, fecal examinations, and drafting of the manuscript. WB-S: cell culture and oocyst manipulation. RJ: cell culture and microscopic analysis. LG: designed the experiment and revised the manuscript. All authors revised the manuscript and approved its final version.

### Conflict of Interest

The authors declare that the research was conducted in the absence of any commercial or financial relationships that could be construed as a potential conflict of interest.
